# Aggregation of Whey Protein Hydrolysate Using Alcalase 2.4 L

**DOI:** 10.1371/journal.pone.0109439

**Published:** 2014-10-07

**Authors:** Chunhong Liu, Wen Liu, Zhibiao Feng, Dongmei Li

**Affiliations:** Department of Applied Chemistry, College of Science, Northeast Agricultural University, Harbin, China; CNR, Italy

## Abstract

Here, we describe peptide aggregation, which is also known as enzymatic protein resynthesis. Whey protein hydrolysate (WPH) is the starting material for assembling peptides. Analyses of the involved amino acids, intrinsic fluorescence, fluorescence phase diagram, secondary structure, turbidity, and surface hydrophobicity were performed to investigate the reaction process. The aggregation mechanism consists of two parts: 1) formation and 2) aggregation of the building blocks that form the ordered secondary β-sheet structure. Constructing the building blocks requires at least one intermediate state, which is formed after 0.5 hours. Non-synergistic changes in the secondary and tertiary structures then allow the intermediate state to emerge.

## Introduction

Protein hydrolysates can aggregate into high-molecular substances and/or less soluble substances. Some chemists initially called this reaction the “plastein reaction”, which was characterized by enzymatic protein degradation and resynthesis [1]. Guo et al. reported that β-conglycinin could form soluble aggregates, but the association of glycinin with the aggregates led to the appearance of insoluble materials [2]. Some aggregates demonstrate amorphous organization [3], whereas others form highly ordered amyloid-like fibrils [4]-[6]. These findings suggest that protein aggregation may be a common structural property of proteins and a much more widespread event than previously suspected.

In food industry applications, these aggregating protein hydrolysates can incorporate limited amino acids into polypeptides, remove the bitter taste of protein hydrolysates, and improve the nutritional value and functional properties of food proteins [7]-[11]. Therefore, aggregation reactions have enormous potential applications in industry.

Although peptide aggregation has been researched for a long time, researchers have not reached a consensus. Yamashita et al. considered the condensation reaction was the main aggregation mechanism [12]. Edwards & Shipe reported that aggregates were held together by noncovalent bonds, rather than covalent peptide bonds and they concluded that physical forces such as hydrophobic interactions play a major role [13]. Andrews performed aggregate reactions on casein hydrolysate and reported that the reaction was an entropy-driven physical aggregation process [14]. The results of Yuan et al. indicated that the soluble soy protein isolate (SPI) - chitosan (CS) aggregate was driven by electrostatic interaction [15]. Ringgenberg et al. concluded that short range interactions play a major role in the formation of the protein network in soymilk curd [16].

The aim of the present study is to research the factors associated with peptide aggregation. It is important to determine a unified and clear process, which will be of great value to further improving protein hydrolysate applications. Here, we study the mechanisms of peptide aggregation by investigating the reaction process, instead of the aggregated product. We believe this protocol may also prove useful in peptide aggregation.

## Materials and Methods

### Reagents

Alcalase 2.4 L was purchased from Novozymes (Bagsvaerd, Denmark). 1-anilinonaphthalene-8-sulfonate (NAS) was purchased from Fluka (Switzerland). All other chemicals and reagents used in this study were analytical grade.

### Whey protein hydrolysate

Whey protein was dissolved in water to a protein concentration of 5% (w/w), and Alcalase 2.4 L was added at an enzyme/substrate (E/S) ratio of 7% (w/w). Hydrolysis was carried out at 50°C for 5 hours. The pH of the hydrolysis system was kept at pH 9 ± 0.05 by continuously adding 1 mol/L NaOH during hydrolysis. The enzyme was inactivated by heating the hydrolysis system in boiling water for 15 minutes. The pH of the hydrolysis system was adjusted to pH 4.0 by adding 2 mol/L HCl in order to precipitate the non-hydrolyzed protein. The mixture was centrifuged at 1800 *g* for 20 minutes to collect the supernatant, then lyophilized to obtain whey protein hydrolysate (WPH) as the substrate of the aggregation reaction.

### Peptide aggregation

The lyophilized WPH powder was dissolved in water to a protein concentration of 30% (w/w), and then the solution was adjusted to pH 7.0. Alcalase 2.4 L was added at an E/S ratio of 2% (w/w) and incubated at 37°C for 0.1, 0.25, 0.5, 1, 2, 3, 4 or 6 hours. The enzyme was inactivated by heating the hydrolysis system in boiling water for 10 minutes. After the reaction was completed, the solution was centrifuged at 11,100 *g* at 4°C for 30 minutes. The synthetic product was then vacuum freeze-dried.

### Amino acid analysis

Amino acids were analyzed according to the professional standards of the People's Republic of China (GB/T14965-1994) [17]. The sample was hydrolyzed for 22 hours at 110°C with 6 mol/L HCl in sealed glass tubes filled with nitrogen. The amino acid concentration in the hydrolyzed samples was diluted to 50 nmol/L using 0.2 mol/L sodium citrate buffer (pH 2.2). The pH-adjusted samples were analyzed using a Biochrom 20 Automatic Amino Acid Analyzer (GE, USA).

### Intrinsic fluorescence analysis

The synthetic products were dissolved in phosphate buffer solution (pH 7.0) and 0.5 mmol/L sodium dodecyl sulfate (SDS) to a protein concentration of 1% (w/w). The liquid was homogenized at 8000 rpm for 1 minute, and then centrifuged at 1500 *g* for 10 minutes. The supernatant was collected by filtering through a 0.45-µm cellulose acetate membrane, then diluted 100× with phosphate buffer solution for use as a stock solution. The fluorescence spectrum was measured using the PE LS-55 Fluorescence Spectrophotometer (Perkin Elmer). Each scan was repeated 5× at a 295 nm excitation wavelength, 10 nm emission slit width, 10 nm excitation slit width, 1200 nm/minute scanning speed, and 300–450 nm scan range.

### Measurement of the infrared spectra and data manipulation

Fourier transform infrared (FTIR) spectra were measured using a Tensor 27 FTIR spectrometer (Bruker, Germany) at a resolution of 4 cm^−1^, and 64 scans were obtained between 400–4000 cm^−1^. The system was continuously purged with N_2_. Reference spectra were recorded under identical conditions, except the KBr media contained no protein.

Second derivative spectra and Fourier deconvolution spectra (18 cm^−1^ full width at half maximum [FWHM]; 2.8 enhancement factor) were determined using OPUS 6.0. By curve-fitting the deconvolution spectra, multiple iterations were constructed to ensure that the residual mean square (RMS) was <0.01.

### Secondary structure analysis of the aggregation reaction process

Accurately weighed samples of potassium ferrocyanide (3 mg; internal standard) and the synthetic products (10 mg; obtained at a different incubation times) were mixed to produce the FTIR spectra. The integrated areas of the amide I band (1595–1705 cm^−1^) and internal standard band (1944–2132 cm^−1^) were determined on the infrared spectra.

### Turbidity analysis

The synthetic product solution was diluted 20× with phosphate buffer solution (pH 7.0), and absorbance at 420 nm was measured using UV-2500 spectrophotometer (Shimadzu, Japan). All measurements were performed in triplicate. Distilled water comprised the blank sample.

### Determination of surface hydrophobicity

ANS (8-anilino-1-naphthalenesulfonic acid)-based measurement of surface hydrophobicity is the most appropriate way to assess proteins and determine the overall three-dimensional structure in solution [18].

The synthetic products were gradually diluted to 0.005–0.1% with 0.1 mol/L phosphate buffer (pH 7.0). Aliquots of the solution (5 mL) were added to 50 µL ANS solution (8 mmol/L ANS and 0.01 mol/L phosphate buffer at pH 7.0) and allowed to stand in the dark for 3 minutes. The fluorescence spectra were obtained using a LS-55 spectrofluorophotometer (Perkin Elmer). The excitation wavelength was 338 nm, and the emission wavelength was 496 nm. The protein concentration was determined using Folin phenol reagent according to the Lowry method [19]. Surface hydrophobicity can be calculated from the initial slope of the fluorescence intensity curve following protein concentration.

## Results and Discussion

### Amino acid analysis

The amino acid compositions of WPH and the aggregates were investigated after incubation for 0.5 or 12 hours, respectively. Table 1 shows the different amino acid compositions of WPH and the aggregates, while the aggregates that were incubated for 0.5 and 12 hours were almost the same. If the aggregation reaction was simply a gradual process, then the amino acid composition of the products in the initial stages of the reaction should be similar to WPH and different from the products identified in the later stages. However, the actual situation was different. We surmise that some basic building blocks are formed in the initial stages; in the latter stages, these building blocks aggregate together to form high-molecular substances.

**Table 1:** Amino acid composition of WPH and aggregates (incubated for 0.5 or 12 h).

Amino acid	Amino acid content(%)		
	WPH	Aggregates (0.5 h)	Aggregates (12 h)
Asp	10.565	10.523	10.365
Thr	6.893	6.954	7.346
Ser	4.022	4.098	4.17
Glu	16.117	13.343	13.256
Gly	2.205	1.972	1.91
Ala	5.687	4.359	5.562
Val	5.846	6.441	6.712
Met	1.664	2.873	3.413
Ile	7.18	8.631	8.862
Leu	9.606	13.248	13.096
Tyr	2.285	2.376	2.437
Phe	2.296	2.397	2.483
His	2.187	2.137	2.093
Lys	6.835	5.46	4.736
NH_4_	3.76	3.074	2.505
Arg	1.991	2.583	2.514
Pro	6.844	6.138	7.122
Trp	Not detected	Not detected	Not detected

### Intrinsic fluorescence

**Figure 1:**
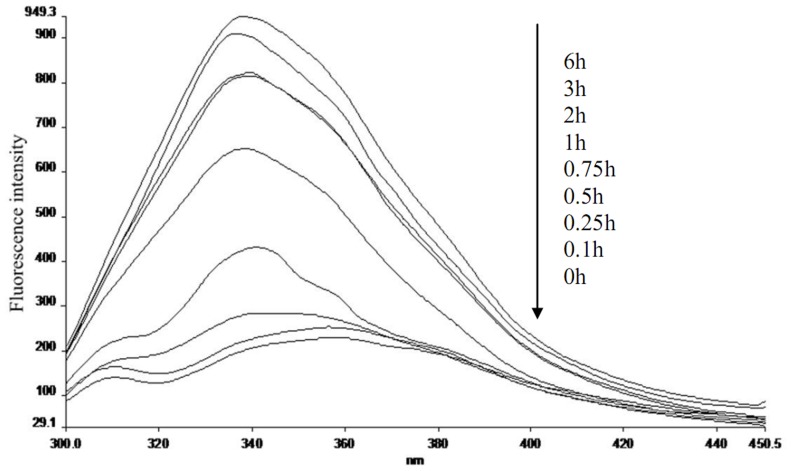
Intrinsic fluorescence spectra of aggregates according to incubation time.

Figure 1 shows the intrinsic fluorescence of the aggregates according to incubation time. Following aggregation, the maximum emission wavelength (λ_em, max_) of the aggregates shifted from 357.98 nm to 340 nm. This blue-shift can be explained by the microstructure, which consists of loose WPH peptide chains that gradually tend toward nonpolar microenvironments. Chromophore amino acid residues were embedded inside the molecule, and the loose WPH peptide chains gradually aggregated to form the hydrophobic regions that constitute the spatial structure.

**Figure 2:**
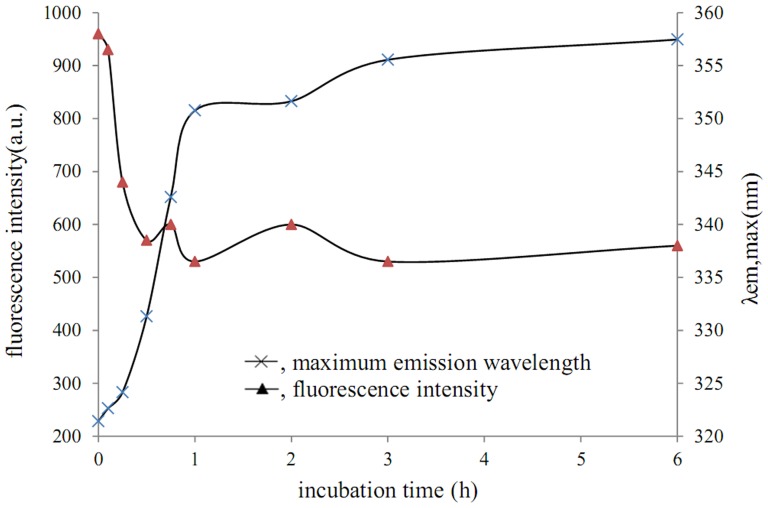
Fluorescence intensity and maximum emission wavelength of aggregates obtained at different incubation times.

Figure 2 shows fluorescence intensity and λ_em, max_ of the obtained aggregates according to incubation time. The rapid increase in fluorescence intensity was observed in the initial stage (0–1 hours), and during this stage λ_em, max_ quickly blue-shifted to 340.72 nm. Fluorescence intensity slowly increased through 6 hours, and λ_em, max_ remained essentially constant at about 340 nm. Some building blocks with hydrophobic cores and stable conformations formed between 0–1 hours. Only the building blocks aggregated together during later stages, and thus no additional new building blocks were formed.

### Fluorescence phase diagram

**Figure 3:**
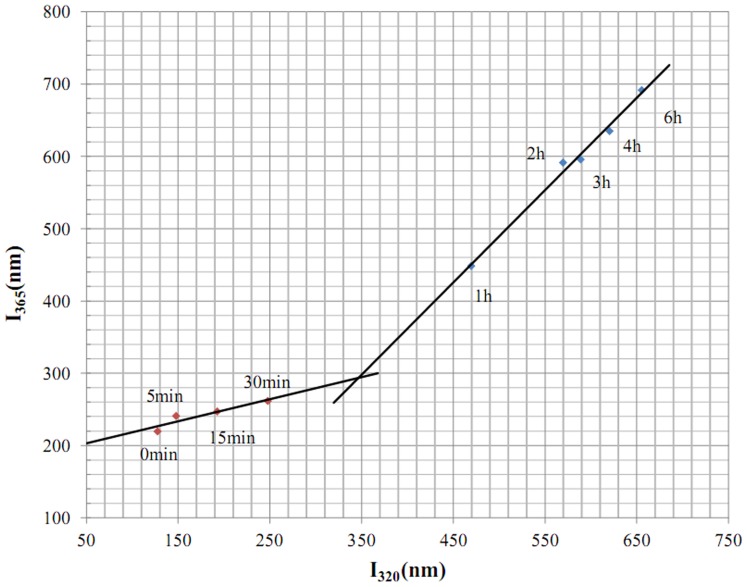
Phase diagram of the aggregates according to incubation time.

Figure 3 shows the phase diagram of the aggregation reaction process. The phase diagram consists of two straight lines with different slopes that intersect at 0.5 hours. This indicates that the aggregate formation process agrees with the three-state model [20]. To conveniently describe the course, the three-state model can be expressed as H, I, and P (H represents the loose polypeptide chains in WPH, I represents the intermediates [i.e., building blocks], and P represents the aggregates). We conclude that there is an intermediate state in the aggregation reaction process, and transitioning from H to I only requires 0.5 hours.

### Secondary structure of the aggregates

**Figure 4:**
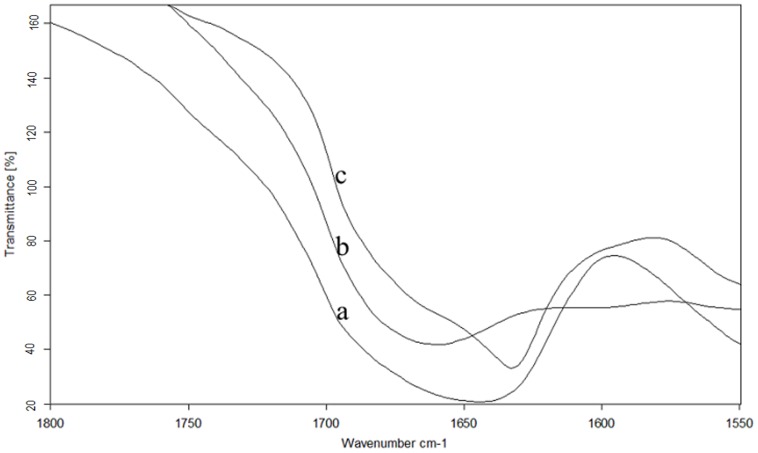
Infrared spectra of the proteins, 400–4000 cm^−1^. a) WPI. b) WPH. c) Aggregates. The system was continuously purged with N_2_.

**Figure 5:**
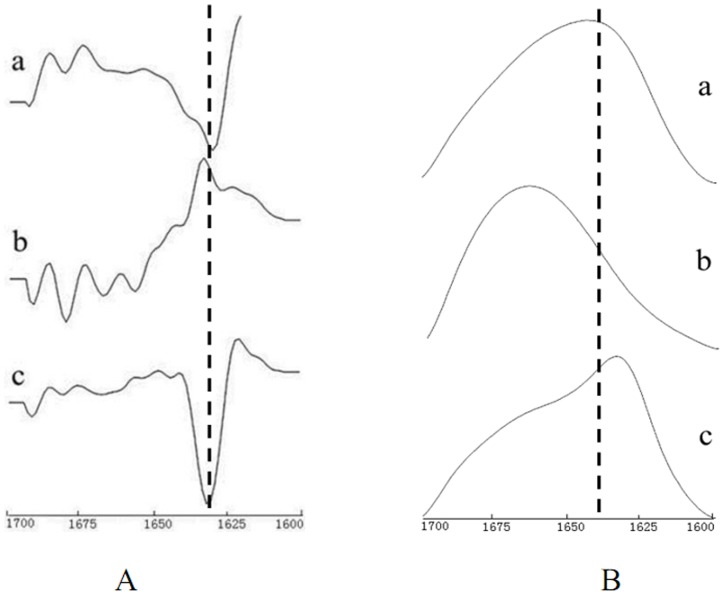
Second-derivative Fourier transform infrared spectra of the protein (A). Deconvolved amide I bands (B). a) WPI. b) WPH. c) Aggregates. (FWHM  =  18 cm^−1^; enhancement factor  =  2.8).

Figure 4 shows the infrared protein spectra of WPI, WPH, and the aggregates. Figure 5A shows the second-derivative Fourier transform infrared spectra of the proteins, and Figure 5B shows the deconvolved amide I bands. Second-derivative analysis of infrared spectra (IR-SD) was used to directly and quantitatively analyze the secondary structural components of proteins, and this technique is considered reliable [21]-[23]. The secondary structure of WPI, WPH, and the aggregates were analyzed using IR-SD. Figure 5A shows how the β-sheet absorption (1638 cm^−1^, 1630 cm^−1^) of WPI disappeared in comparison with WPH, while the random coil (1649 cm^−1^) and β-turn absorption bands (1668 cm^−1^, 1681 cm^−1^) became even more apparent. After incubation, WPH aggregated together to form proteins, and a sharp peak with a wide half-width appeared at 1631 cm^−1^ in the second-derivative spectra. We conclude that WPH barely has an ordered structure, but incubation rearranges the loose peptide chains that form the ordered secondary structure, which mainly consists of β-sheets.

**Figure 6:**
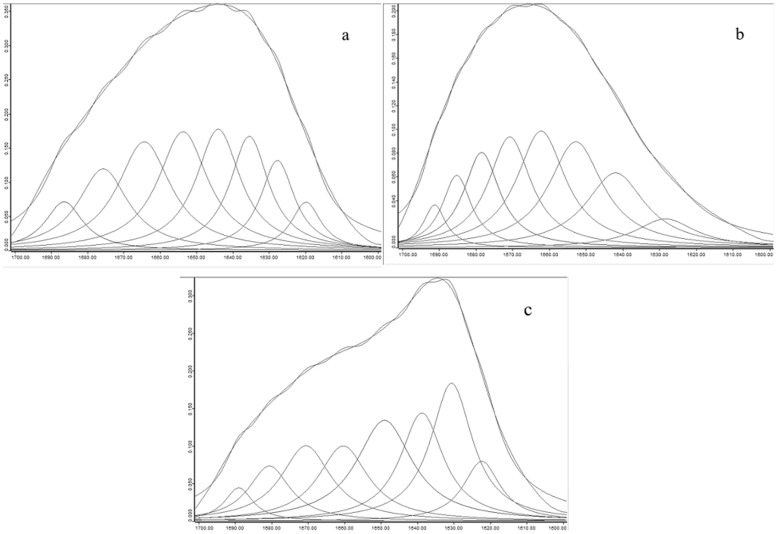
Infrared spectra of the amide I band with curve fitting before and after aggregation. a) WPI. b) WPH. C) Aggregates.

The amide I portion of the spectrum, after deconvolution, is shown in Figure 5B. The peak of the amide I band also initially demonstrated red-shift and then blue-shift. Curve fitting was performed and Gaussian-shaped bands for the deconvolved components were assumed (Figure 6); the curve-fitting results are shown in Table 2. We conclude that the aggregates formed ordered secondary structures [24], indicating that the aggregation reaction was not a messy irregular aggregation but a relatively regular polypeptide self-assembly process. This is different from Otte et al's results [25]. Otte et al. studied aggregation formation in the hydrolysis process, and found that the secondary structure gradually disappeared with aggregation formation.

**Table 2:** Curve-fitting results.

Secondary structure	WPI		WPH		Aggregates	
	(cm^−1^)	(%)	(cm^−1^)	(%)	(cm^−1^)	(%)
α-helix	1653	18.61	1653	19.91	1658	12.99
β-sheet	1619	40.67	1628	21.21	1623	53.64
	1628		1678		1631	
	1635		1691		1638	
	1675				1690	
Unordered	1644	16.79	1642	15.43	1649	19.39
β-turns and bends	1664	23.91	1662	43.43	1671	33.92
	1686		1671		1681	
			1685			

### Secondary structure analysis of the aggregation reaction process

**Figure 7:**
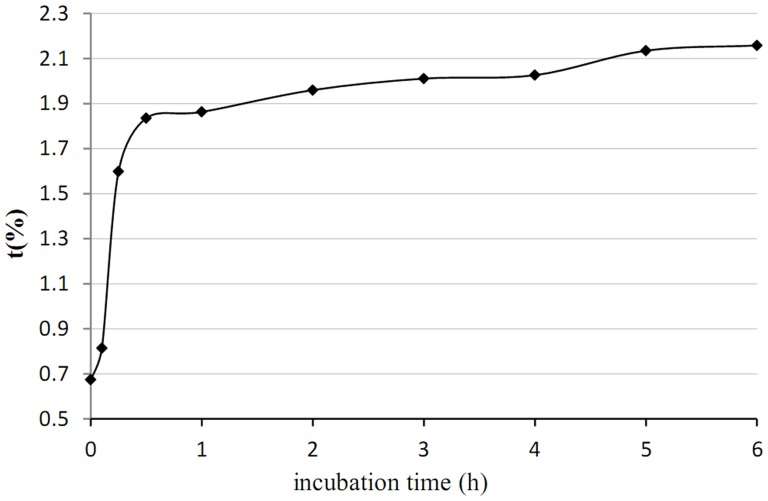
t (%) value of aggregates obtained at different incubation times.

The fate of the secondary structure during aggregation was investigated using the infrared internal standard method. The above discussion gives some justification for the assumption that aggregation leads to structural rearrangement; this also means the disordered WPH structure becomes a well-ordered structure of aggregates that mainly consists of β-sheet structures. β-Sheets possess considerable hydrogen bonds, particularly ordered hydrogen bonding and the orderly arrangement of infrared activity in specific regions in amide I. The regular and orderly arrangement of hydrogen bonds could only occur in the ordered secondary structures (α-helix and β-fold). Therefore, following aggregate synthesis, an ordered structure gradually forms under the interacting hydrogen bonds and the corresponding narrow absorption bands appear in specific regions of amide I. The area of the absorption peak will also gradually increase.

t (%) is the ratio of the peak area of the amide I band to the internal standard. Because the band of the internal standard is fixed, any increase in t (%) represents an increase in the band area of amide I (i.e., an increase in the ordered secondary structure) [26], [27]. Figure 7 shows the t (%) values of the aggregates that were obtained at different incubation times. The rapid increase in t (%) was observed in the initial stage (0–0.5 hours), and then a very slow increase in t (%) occurred after 0.5 hours.

### Turbidity and surface hydrophobicity

**Figure 8:**
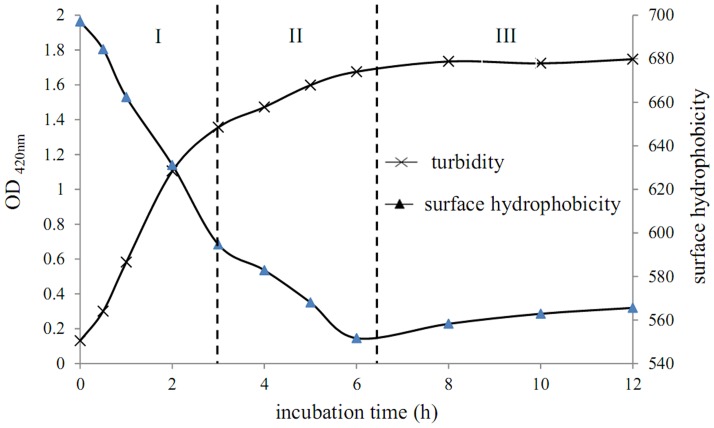
Turbidity and surface hydrophobicity of aggregates obtained at different incubation times.

Aggregates were gradually synthesized during incubation with Alcalase 2.4 L (Figure 8), and turbidity and surface hydrophobicity also gradually changed. Both trends can be divided into three parts: 0–3 hours, 3–6 hours, and > 6 hours. The reaction rates were fast, medium, and slow, respectively. This means that the aggregation processes described are the same.

The changes in surface hydrophobicity also illustrate the hydrophobic interactions of the reaction processes. At 0–3 hours, surface hydrophobicity fell very fast due to the strong hydrophobic interactions generated by the accumulation of hydrophobic side chains. Surface hydrophobicity further declined over 3–6 hours and the reaction speed slowed, indicating that aggregation approached equilibrium. Finally, at 6–12 hours, surface hydrophobicity slightly increased because after achieving equilibrium because additional noncovalent bonds formed inside the aggregate, which led to a more compact aggregation structure as the hydrophobic regions became embedded.

Speculation regarding a “small structures group” is based on the assumption of “basic building blocks” in the construction of amino acids [28]. Intrinsic fluorescence spectra analysis shows that 1 hour is the turning point for structural changes and when structural groups form. Small structures have a stable spatial structure and hydrophobic core, in addition to the ordered secondary β-sheet. An intermediate structure was identified at 0.5 hours on the phase diagram. At the same time, the turning points for λ_em,max_ and t (%) occurred at 0.5 hours. The demarcation point at 0.5 hours indicates non-synergy, and the endogenous fluorescence spectrum changes in terms of fluorescence intensity and λ_em,max_. Taking into account the non-synergistic changes between fluorescence intensity and λ_em,max_, we conclude that the intermediate appeared during the information of the small structures group because of non-synergy between the secondary and tertiary structures. In other words, the secondary structure forms prior to the tertiary structure; furthermore, when the secondary structure is formed, the tertiary structure is still being built. Therefore, this intermediate has a complete secondary structure and a fairly complete hydrophobic core.

## Conclusion

Aggregation consists of two parts according to the results of the amino acid analysis, intrinsic fluorescence spectra, and infrared spectra. First, building blocks with hydrophobic cores and a stable conformation form between 0–0.5 hours. After 0.5 hours, the building blocks aggregate together and no additional new building blocks form. The small structures group is involved in aggregation, and this process can be divided into three parts: fast, medium, and slow.

The second-derivative Fourier transform infrared spectra and deconvolved amide I band infrared spectra of the proteins confirm that the aggregates demonstrate a stable spatial structure, hydrophobic core, and ordered secondary β-sheet structure relative to the reaction substrate. Hydrophobic interactions and hydrogen bonds play an important role in aggregation.
